# Minimizing *N*-Nitrosodimethylamine Formation During Disinfection of Blended Seawater and Wastewater Effluent

**DOI:** 10.1021/acsestwater.3c00617

**Published:** 2024-02-06

**Authors:** Sophia L. Plata, Amy E. Childress, Daniel L. McCurry

**Affiliations:** Astani Department of Civil and Environmental Engineering, University of Southern California, Los Angeles, California 90089, United States

**Keywords:** potable reuse, desalination, *N*-nitrosodimethylamine formation, disinfection byproducts

## Abstract

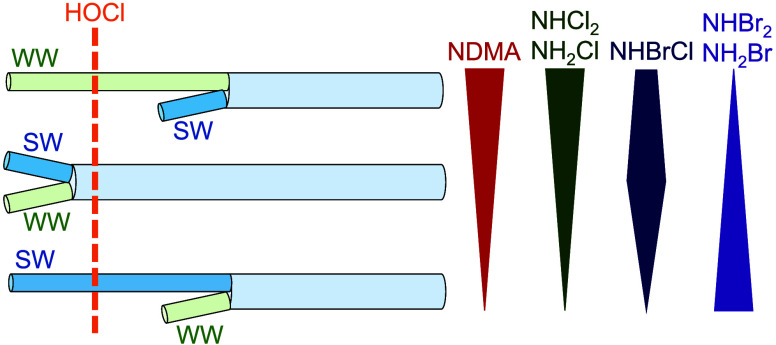

Augmenting seawater with wastewater has the potential to reduce the energy demand and environmental impacts associated with seawater desalination. Alternatively, as wastewater reuse becomes more widespread, augmenting wastewater with seawater can increase the available water supply. However, the chemistry of disinfecting a blended stream has not been explored. Toxic byproducts, including *N*-nitrosodimethylamine (NDMA), are expected to form during disinfection, and the extent of formation will likely be a function of which stream is chlorinated and whether disinfection happens before or after blending. In this work, three blending-disinfection scenarios were modeled and experimentally evaluated in bench-scale systems treating synthetic and authentic waters. Modeling results suggested that chlorinating preblended wastewater and seawater would produce the most NDMA because it yielded the highest concentrations of bromochloramine, which was previously found to promote NDMA formation. However, chlorinating wastewater prior to blending with seawater, which modeling indicated would form the most dichloramine, produced the most NDMA in experiments. When seawater was disinfected prior to blending with wastewater, bromide likely converted most chlorine to free bromine. Bromamines formed after blending, however, did not lead to an elevated level of NDMA formation. Therefore, to minimize NDMA formation when disinfecting blended wastewater–seawater, seawater should be disinfected prior to introducing wastewater.

## Abstract 2

This study evaluated NDMA formation in three potential blending and disinfection scenarios for blended synthetic and authentic seawater and wastewater. Chlorinating seawater prior to blending with wastewater and limiting the wastewater contribution decreased NDMA formation.

## Introduction

The deliberate blending of seawater and wastewater effluent for potable use has not been extensively studied, despite increased use of both individually as potable water supplies. Currently, the unintentional blending of seawater and wastewater effluent occurs as a result of seawater intrusion and infiltration that has been observed at coastal wastewater treatment facilities due to aging infrastructure, corroded pipes, and leaky gates.^1−3^ While the contribution of dissolved solids from seawater (with a total dissolved solids concentration of ∼35,000 mg/L) may complicate wastewater treatment and reuse, it also slightly increases the volume of water available for meeting potable water demand. At seawater desalination facilities, introducing wastewater effluent into intake seawater could reduce the cost and environmental impacts associated with seawater desalination by reducing pumping energy requirements and waste brine salinity.^4−7^

**Figure 1:**
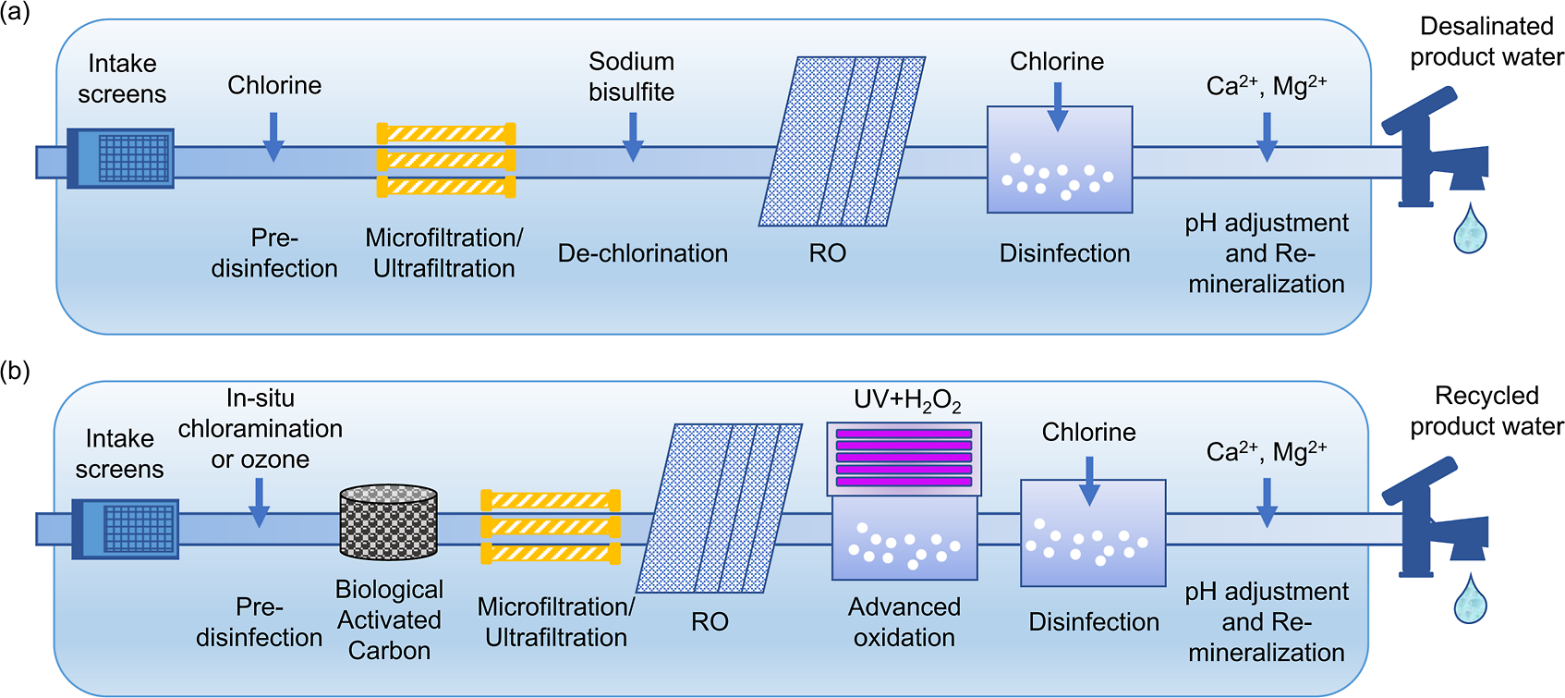
Simplified treatment trains of a (a) seawater desalination facility and (b) potable reuse facility.

A recent energy feasibility study on treating blended seawater and wastewater effluent streams highlighted significant similarities between typical desalination and potable reuse trains.^6^ Both treatment trains typically employ a microfiltration or ultrafiltration step followed by reverse osmosis (RO); however, they differ in their disinfection and oxidation processes. Different predisinfectants are applied upstream of the membrane processes, and an ultraviolet light advanced oxidation process (UV/AOP) follows RO in typical potable reuse treatment trains (Figure 1).

Although the blending of seawater and wastewater streams could have energetic, economic, and environmental benefits, the chemistry of disinfecting a blend of seawater and wastewater has not been studied. In particular, bromide from seawater and ammonia from wastewater (either naturally present or added as part of the chloramination process) may alter the speciation of disinfectants applied for pathogen and fouling control, likely affecting the formation of disinfection byproducts (DBPs) compared to free chlorination in the absence of ammonia or bromide. Therefore, the addition of oxidants and/or disinfectants to a blended stream merits careful consideration.

The most commonly used predisinfectant during seawater desalination is free chlorine (HOCl/OCl^–^).^8−10^ Seawater chlorination produces free bromine (HOBr/OBr^–^) by rapidly oxidizing bromide (*k*_HOCl,Br^–^_ = 1.55 × 10^3^ M^–1^ s^–1^Table S1),^11^ and free bromine is primarily responsible for disinfection and oxidation in chlorinated seawater.^12^ Compared to free chlorine, free bromine disinfection is effective over a wider range of pH values,^13^ and generally reacts much faster with organic compounds.^14^ However, chlorination of high-bromide waters, such as seawater, is associated with the formation of brominated DBPs,^15−17^ which are more cytotoxic and genotoxic than chlorinated analogues.^18−21^

Predisinfection of wastewater effluent for potable reuse is most commonly achieved with chloramines [a mixture of monochloramine (NH_2_Cl), and trace amounts of dichloramine (NHCl_2_) and trichloramine (NCl_3_), produced by reactions between free chlorine and ammonia (Table S1)].^22,23^ Chloramines tend to form lower concentrations of halogenated DBPs compared to free chlorine,^24^ and are less destructive to the surface of polyamide membranes used for RO;^25−27^ however, chloramination of wastewater effluent is associated with the formation of NDMA.^28,29^ NDMA is a probable carcinogen,^30^ and while not federally regulated in drinking water, California has established a Public Health Goal of 3 ng/L, and a notification level of 10 ng/L.^31^ NDMA is also on the US EPA Candidate Contaminant List 5 for federal regulatory consideration,^32^ is federally regulated in Canada with a maximum acceptable concentration of 0.04 μg/L in drinking water,^33^ and is recognized as a potent carcinogen by the World Health Organization with an established guideline value of 0.1 μg/L in drinking water.^34^ NDMA formation led to the closure and retrofit of an early water reuse system,^35,36^ and most modern reuse facilities employ UV/AOP, in part for NDMA control.^37^ Because NDMA abatement by UV/AOP is not always complete and effluent concentrations are proportional to concentrations entering the UV/AOP system,^38^ minimizing NDMA formation during disinfection remains useful. NDMA in wastewater is thought to form primarily during reactions between chloramines and organic amine precursors. The most commonly studied model NDMA precursors are secondary amines (e.g., dimethylamine,^29,39−42^ which forms NDMA at ∼2–8% yield depending on chloramine speciation) and tertiary amines, especially *N*,*N-*dimethyl-α-arylamines (e.g., ranitidine,^43,44^ or dimethylbenzylamine^45,46^) which form NDMA at molar yields approaching 100%.

While the use of free chlorine has been widely examined in both seawater desalination^47,48^ and potable reuse,^49−51^ chloramines have only been extensively studied in the context of water reuse and drinking water treatment,^29,52−54^ and not in seawater desalination. When seawater (containing bromide) is chloraminated, it is expected to form bromamines [monobromamine (NH_2_Br), dibromamine (NHBr_2_), and bromochloramine (NHBrCl)], whose reactivity is less well-studied than chloramines. NHBrCl, the principal product formed from monochloramine and free bromine,^55^ has been proposed as the species responsible for promoting NDMA formation in chloraminated waters containing bromide,^28,55−59^ by reacting with amines to form brominated hydrazine intermediates, which are further oxidized to form NDMA.^28,55^ Therefore, we suspected that chlorinating water containing both bromide and ammonia might lead to greater NDMA formation than water containing only bromide or ammonia.^60^

In this work, the kinetics and speciation of chloramines and bromamines formed during the blending and disinfection of seawater and wastewater for potable reuse applications were studied. Halamine speciation (i.e., chloramine and bromamine) was computationally modeled under three blending/disinfection scenarios and three wastewater-to-seawater ratios. Halamine formation and speciation were expected to depend on whether the seawater or wastewater stream is chlorinated prior to blending with the other stream or chlorine is added after blending. Dichloramine and bromochloramine concentrations were examined especially closely, as these species have previously been associated with NDMA formation. Halamine kinetics modeling was followed by bench-scale disinfection experiments using synthetic and authentic seawater and wastewater effluent to determine which blending and disinfection sequence produced the least NDMA, and whether changes to NDMA formation as a function of the disinfection approach were consistent with differences in modeled halamine speciation under the same conditions.

## Materials and Methods

### Disinfectant Speciation Modeling

Halamine concentrations were modeled in Kintecus,^52^ using reaction rate constants (Table S1) and representative concentrations of relevant species in seawater and secondary wastewater effluent (Table S2) from the literature. Reported wastewater–ammonia concentrations ranged from 0.3–62 mg/L, and averaged 12 mg/L. In this study, 10 mg/L was used for modeling and bench-scale experiments. Additional details and references on seawater and wastewater composition are provided in Tables S3 and S4. Halamine speciation was determined computationally rather than experimentally because chloramine concentrations are typically determined spectrophotometrically by measuring simultaneous absorbance at the absorption maxima of monochloramine and dichloramine and solving two simultaneous equations to obtain their respective concentrations. However, the addition of bromide permits the formation of at least five halamine species, whose absorbance spectra might substantially overlap. The disinfectant speciation model was validated by replicating experimental and modeled speciation results from previous studies (Figures S1 and S2).^58,61^

**Figure 2:**
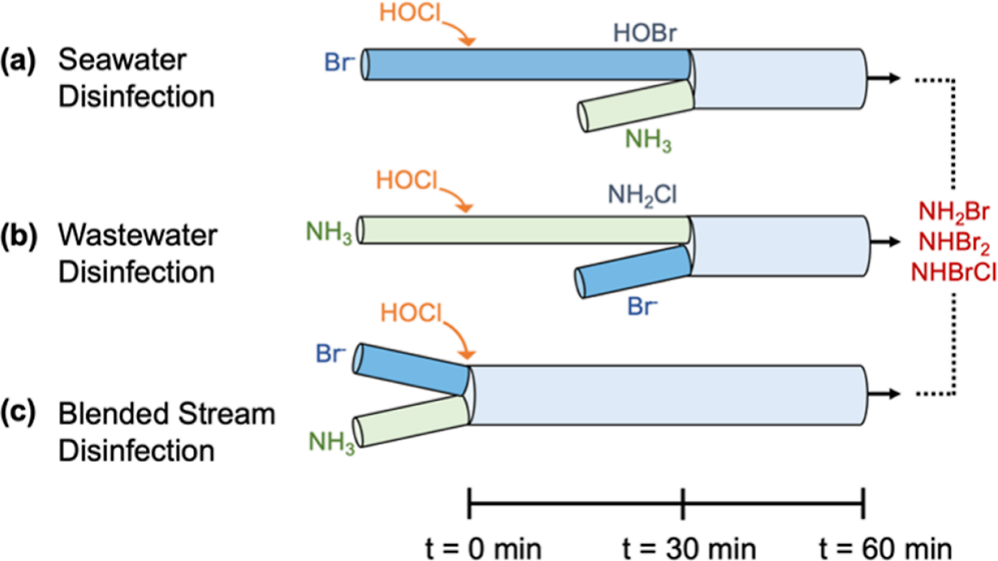
Three blending/disinfecting strategies in which (a) seawater is disinfected alone before the addition of wastewater, (b) wastewater is disinfected alone before the addition of seawater, and (c) the blended stream is disinfected after blending.

Disinfectant speciation was modeled for three blending/disinfecting scenarios: (a) chlorinating seawater prior to blending with wastewater, (b) chlorinating wastewater effluent prior to blending with seawater, and (c) chlorinating combined wastewater effluent and seawater after blending. Blending occurred prior to disinfection (*t*_rxn_ = 0 min) in the preblended stream, and halfway through the chlorine contact time (*t*_rxn_ = 30 min) when chlorine was added to one or the other stream prior to blending (Figure 2). Speciation was modeled for 60 min of simulated contact chlorine time. In a future blended stream treatment operation, variable amounts of seawater could be blended with a base flow of wastewater to adjust to variable water demand.^56^ Therefore, three blending ratios were evaluated: (i) 25% wastewater and 75% seawater, (ii) 50% wastewater and 50% seawater, and (iii) 75% wastewater and 25% seawater for each blending/disinfecting scenario.

### Materials and Reagents

All salts and buffers used to prepare synthetic seawater and wastewater for bench-scale experiments (Table 1), were purchased at the highest available purity from Sigma-Aldrich (St. Lous, MO). Model NDMA precursors [dimethylbenzylamine (DMBzA) and dimethylamine (DMA)] were also purchased at the highest available purity from Sigma-Aldrich. Standard solutions of NDMA and *d*_6_-NDMA (1000 μg/L in methylene chloride) were purchased from AccuStandard (New Haven, Connecticut) and diluted in methyl *tert*-butyl ether to prepare spiking solutions.

**Table 1:** Compositions of Seawater and Wastewater Effluent, Including Ionic Strength (μ) and pH, Used for Modeling and Creating Synthetic Streams for Bench-Scale Experiments^a^

stream	chlorine (M)	bromide (M)	chloride (M)	[PO_4_]_TOT_(M)	[NH_3_]_TOT_(M)	μ(M)	pH
seawater	1.41 × 10^–5^	7.88 × 10^–4^	5.36 × 10^–1^	0.01	5.87 × 10^–7^	0.70	8.10
wastewater	7.05 × 10^–5^	1.25 × 10^–6^	4.65 × 10^–3^	0.01	5.88 × 10^–4^	0.01	7.50
blend (1:1 v/v)	4.23 × 10^–5^	3.95 × 10^–4^	2.70 × 10^–1^	0.01	2.94 × 10^–4^	0.36	7.88

a Values were averages taken from Tables S2–S4.

### Bench-Scale NDMA Formation Experiments

Bench-scale experiments were conducted with synthetic and authentic waters in triplicate headspace-free 23.5 mL amber borosilicate glass vials capped with PTFE-faced septa. Synthetic seawater and synthetic wastewater were prepared in 23.5 mL of 10 mM phosphate buffer (diluted from a 1 M stock solution) at pH 7 with the constituents shown in Table 1. Synthetic wastewater was dosed with 50 μM of DMBzA or DMA as model NDMA precursors. Authentic seawater was collected from the influent of a desalination facility and authentic wastewater was collected from the secondary effluent of a wastewater utility, both in Southern California. Water quality data for both waters are available in the Supporting Information (Table S5).

Chlorine was prepared from working stocks of a 5% NaOCl solution (Fisher Scientific) and standardized by UV spectroscopy.^62^ Regardless of which stream was disinfected first, chlorine was dosed such that the nominal free chlorine concentration after blending was 5 mg/L Cl_2_ (e.g., by adding 10 mg/L chlorine to wastewater before blending with an equal volume of seawater or adding 5 mg/L to water blended prior to disinfection). Chlorine dose was chosen based on typical values obtained from the literature (Table S2). Experiments were conducted in the dark at *T* = 22 ± 1 °C. In the seawater disinfection and wastewater disinfection scenarios, blending occurred 30 min after chlorination, and after an additional 30 min, solutions were quenched with 12.5 mg/L of ascorbic acid from a 200 mM stock solution to halt the reaction. The disinfectant was applied as if it were upstream of a membrane process, allowing for a 60 min contact time for the chlorine disinfection process, as opposed to the longer contact times typically used in NDMA formation studies to represent drinking water conditions [e.g., the simulated distribution system (SDS) test].^63−66^ As with halamine speciation modeling, multiple blending ratios were evaluated for NDMA formation in the same manner.

### Analytical Methods

Quenched 23.5 mL samples were extracted with dichloromethane (10% v/v; shaken for two min), and extracts were dried with 1 g of magnesium sulfate. Ten μg/L *d*_6_-NDMA was added to aqueous samples as an internal standard prior to extraction. A relatively high concentration of *d*_6_-NDMA was used because of the small volume extracted (23.5 mL), compared to the typical 500 mL extraction volume used for trace NDMA quantification in drinking water (e.g., EPA Method 521).^67^ NDMA formation was quantified after extraction using gas chromatography and tandem mass spectrometry (GC/MS/MS) (Agilent 7890/7010, Santa Clara, CA). The vials were introduced by an automated liquid sampler (Agilent 7693A) and injected onto a DB-1701 column (60 m × 0.25 mm × 0.25 μm) at 150 °C. The GC oven temperature program began at 40 °C with a 1 min hold, was increased to 150 °C at 10 °C/min, and then held at 250 °C for 1 min. The MS interface temperature was set at 250 °C. Mass spectra of NDMA and *d*_6_-NDMA were acquired in multiple reaction mode with mass transitions of 74 to 44 and 80 to 50 *m*/*z*, respectively. Analytical method performance data, including a sample standard curve, calculated extraction efficiency, and limit of detection determination are provided in Text S1, Table S6, and Figure S6.

## Results and Discussion

### Modeled Dichloramine and Bromochloramine Formation and Exposure as a Function of Blending Strategy

**Figure 3:**
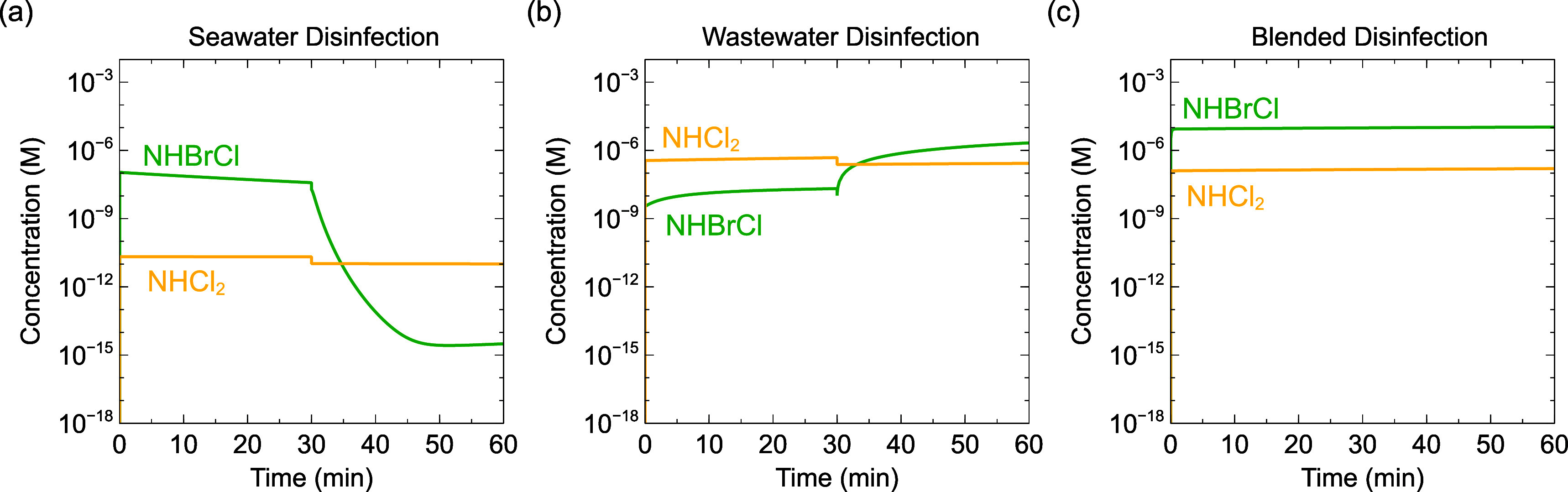
Dichloramine and bromochloramine concentrations modeled as a function of blending scenario: (a) seawater disinfection prior to blending, (b) wastewater disinfection prior to blending, and (c) blended disinfection. All scenarios were blended at equal volumetric ratios of seawater and wastewater using the compositions from Table 1. References are included in Tables S2–S4.

Halamine concentrations were modeled over 60 min of simulated contact time under each of the three blending/disinfection sequences (Figure S3). Cumulative halamine exposures were also estimated (Figure S4). Dichloramine (NHCl_2_) and bromochloramine (NHBrCl) concentrations were of the most interest because they are the species that have been associated with NDMA formation.^28,55,63^ Modeled dichloramine concentrations were lowest when seawater was disinfected before blending with wastewater (Figure 3a) because the conversion of free chlorine to free bromine prior to encountering wastewater–ammonia led to the formation of primarily bromamines after blending (Figure S3). Dichloramine formation was greatest when wastewater was disinfected before blending (Figure 3b). Ammonia in the wastewater was nearly instantly oxidized to monochloramine upon chlorination (*k*_app,pH 7_ = 4.20 × 10^6^ M^–1^ s^–1^).^68^ In disinfected wastewater, dichloramine formed primarily by the chlorine transfer reaction converting two monochloramine molecules to dichloramine and ammonia (d[NHCl_2_]/d*t* = 5.86 × 10^–11^ M/s),^69^ versus the somewhat slower chlorination of monochloramine by free chlorine (d[NHCl_2_]/d*t* = 1.50 × 10^–11^ M/s). When disinfection occurred after blending (Figure 3c), dichloramine formation by both chlorine transfer (d[NHCl_2_]/d*t* = 8.17 × 10^–12^ M/s) and monochloramine chlorination by free chlorine (d[NHCl_2_]/d*t* = 3.50 × 10^–12^ M/s) were somewhat slower. Slower modeled dichloramine formation in blended disinfection than wastewater disinfection likely occurred because excess free chlorine was rapidly depleted by bromide and because some monochloramine reacted with free bromine to form bromamines, as discussed below.

When seawater was chlorinated prior to blending, trace seawater–ammonia was nearly instantaneously oxidized to bromochloramine (Figure 3a). After introduction of wastewater to chlorinated seawater at *t* = 30 min, the modeled bromochloramine concentration rapidly declined as it oxidized the ammonia in the wastewater forming monochloramine and monobromamine (k_NH_3_,NHBrCl_ = 3.90 × 10^2^ M^–1^ s^–1^).^58^ During wastewater disinfection, bromochloramine concentrations increased after blending with seawater (Figure 3b), despite the lack of a direct bromide oxidation pathway for monochloramine (Table S1). Monochloramine was preferentially consumed by free bromine species (produced by bromide reactions with monochloramine leading to free bromine, as discussed further below) to form bromochloramine (*k*_HOBr,NH_2_Cl_ = 2.86 × 10^5^ M^–1^ s^–1^; *k*_Br_2_,NH_2_Cl_ = 4.18 × 10^8^ M^–1^ s^–1^),^70^ outcompeting dichloramine formation (*k*_HOCl,NH_2_Cl_ = 2.78 × 10^2^ M^–1^ s^–1^).^69^ Bromochloramine formed nearly instantaneously during blended disinfection (Figure 3c), and remained higher than in the other two scenarios throughout the reaction time.

**Figure 4:**
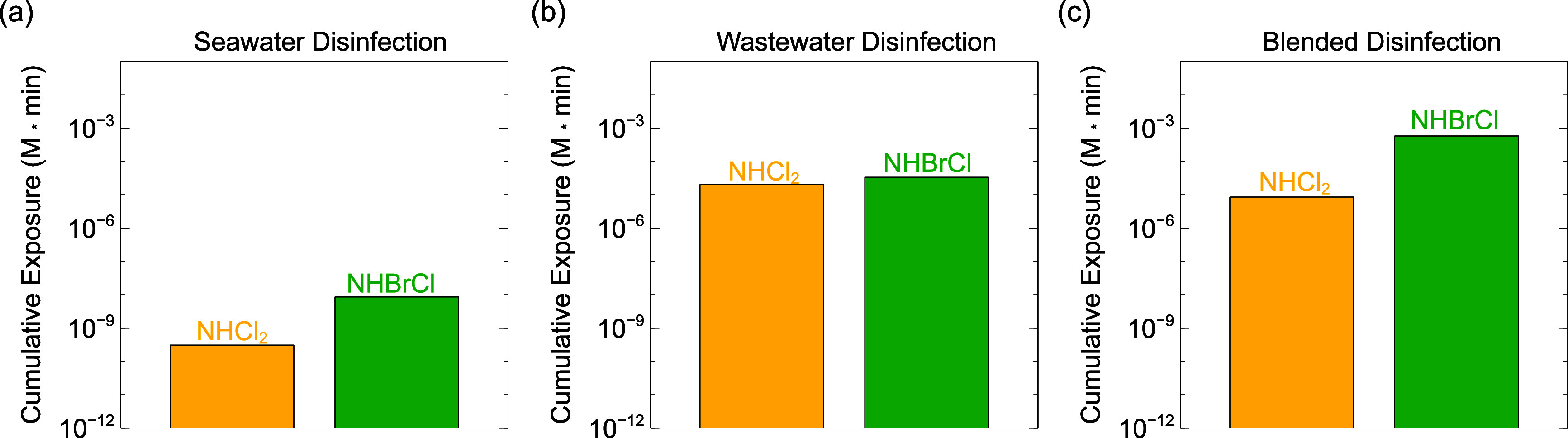
Cumulative dichloramine and bromochloramine exposures (concentration × time) modeled as a function of the blending scenario: (a) seawater disinfection prior to blending, (b) wastewater disinfection prior to blending, and (c) blended disinfection. All scenarios were blended at seawater/wastewater = 1:1 v/v.

In addition to instantaneous concentrations of dichloramine and bromochloramine, cumulative exposures were modeled because NDMA formation kinetics are relatively slow and might take place throughout the disinfection step. During seawater disinfection (Figure 4a), modeled dichloramine exposure was lower than bromochloramine exposure, because most chlorine was consumed by oxidizing bromide to form hypobromous acid before blending (*k*_HOCl,Br^–^_ = 1.55 × 10^3^ M^–1^ s^–1^).^11^ Hence, negligible free chlorine remained when the water encountered wastewater–ammonia to form chloramines. Cumulative exposure to dichloramine was greater during wastewater disinfection and blended disinfection (Figure 4b,c). Dichloramine exposure was slightly higher during wastewater disinfection than blended disinfection, due to competition between ammonia and bromide for chlorine in blended water.^71^

Modeled bromochloramine exposure was lowest during seawater disinfection (Figure 4a), and highest in blended disinfection (Figure 4c), because bromochloramine forms primarily by reactions between monochloramine and free bromine. In blended disinfection, both ammonia and bromide are simultaneously available for reaction with free chlorine. The products of these reactions, monochloramine and free bromine, can then be combined to form bromochloramine.

During wastewater disinfection (Figure 4b), chlorine was rapidly consumed by oxidizing ammonia (*k*_HOCl,NH_3__ = 4.2 × 10^6^ M^–1^ s^–1^)^68^ before encountering seawater bromide. Bromide encountered after blending can react with monochloramine to generate BrCl, which can further react with bromide or hydrolyze, forming free bromine species Br_2_ and HOBr, respectively (Table S1). These free bromine species can then react with monochloramine to form bromochloramine, potentially explaining bromochloramine formation despite the lack of a direct bromide oxidation reaction by monochloramine. However, modeled bromochloramine exposure relative to dichloramine exposure was lowest when chlorinating wastewater prior to blending, likely because a sequence of several reactions is needed to produce bromochloramine from monochloramine and bromide. Because bromochloramine has previously been proposed to explain the enhanced formation of NDMA in the presence of bromide and modeling results predicted higher bromochloramine formation during blended disinfection than seawater or wastewater disinfection, blended disinfection was anticipated to produce the highest concentrations of NDMA, and seawater disinfection was anticipated to produce the least NDMA, as further investigated below.

### Effect of Blending Ratio on Chloramine and Bromamine Speciation during Disinfection

**Figure 5:**
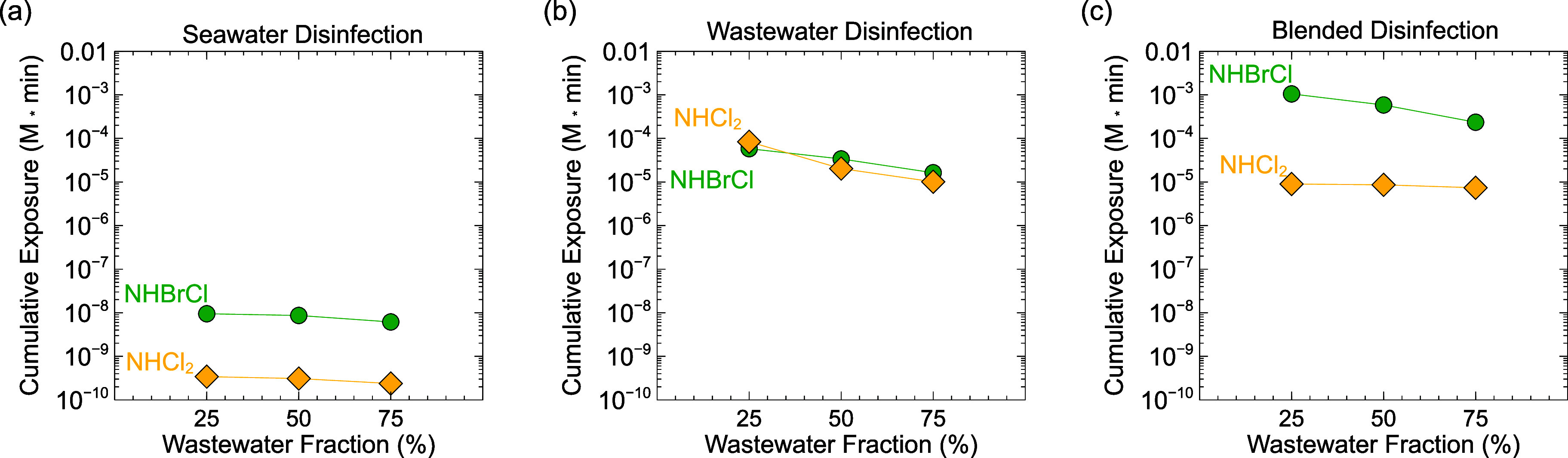
Cumulative dichloramine and bromochloramine species concentrations modeled as a function of wastewater fraction for (a) seawater disinfection prior to blending, (b) wastewater disinfection prior to blending, and (c) blended disinfection.

In each modeled blending scenario, cumulative dichloramine exposure decreased with an increasing wastewater fraction (Figure 5). This was likely due to elevated ammonia in more wastewater-rich streams leading to a lower chlorine-to-ammonia (Cl/N) molar ratio as wastewater fraction increased (0.48 at 25%, 0.24 at 50%, and 0.16 at 75%), as dichloramine formation is promoted by excess chlorine. Exposure to all halamine species slightly declined in more wastewater-rich blends (Figure S5) due to the lower preblending chlorine dose (accounting for subsequent dilution by blending, to achieve the same consistent dose of 5 mg/L final volume), but dichloramine exposure declined more dramatically than monochloramine at elevated wastewater fraction. Smaller bromide concentrations in wastewater-rich blends did not substantially change dichloramine exposure.

Modeled bromochloramine exposure also slightly decreased with an increasing wastewater fraction in all scenarios (Figure 5), likely due to decreased bromide concentrations from reduced seawater volume. In contrast to seawater disinfection or blended disinfection, bromochloramine exposure was not substantially greater than dichloramine exposure in the wastewater disinfection, likely because of the sequence of reactions needed to produce bromochloramine from monochloramine and bromide, as discussed above.

### Effect of Blending Strategy on NDMA Formation

**Figure 6:**
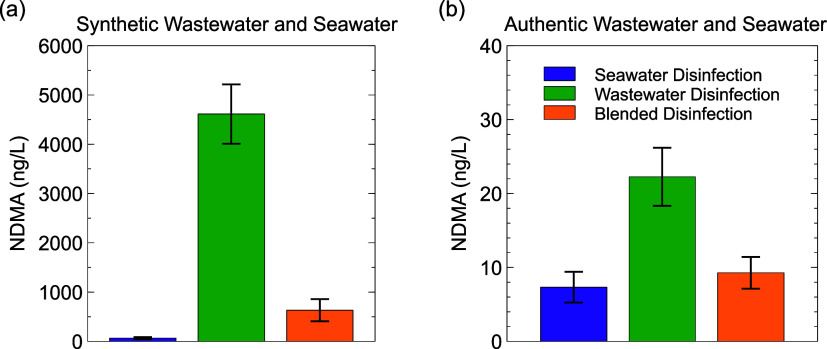
NDMA formation in (a) synthetic seawater and wastewater and (b) authentic seawater and wastewater for seawater disinfection prior to blending (in purple), wastewater disinfection prior to blending (in green), and blended disinfection (in orange). Error bars represent standard deviations of experimental triplicates. Experimental conditions: pH = 7.0, [NaOCl]_0_ = 70.5 μM, [PO_4_]_tot_ = 10 mM, [DMBzA]_0_ = 0.05 mM, *t*_rxn_ = 60 min, *T* = 22 ± 1 °C. Authentic water quality data are available in the Supporting Information (Table S5).

NDMA formation was approximately 2 orders of magnitude greater with synthetic water (Figure 6a) than with authentic seawater and wastewater (Figure 6b) under all blending scenarios, likely because 50 μM of DMBzA was spiked, which can form NDMA at nearly 100% yield,^41^ compared to likely lower concentrations and/or lower average yield from the overall NDMA precursor pool in authentic wastewater. Despite the difference in NDMA levels formed in synthetic and authentic waters, NDMA formation followed similar trends with respect to the blending strategy in both cases. Wastewater disinfection prior to blending formed the most NDMA, while seawater disinfection prior to blending formed the least (Figure 6), although the differences were less pronounced in authentic seawater and wastewater compared to synthetic analogues.

Past mechanistic work,^29,41,42^ and practical experience that minimizing dichloramine minimizes NDMA,^33,72−75^ is consistent with higher NDMA formation when chlorinating wastewater. Simulated wastewater disinfection produced the highest dichloramine concentrations and exposures in the modeling results discussed above. However, elevated NDMA formation in scenarios favoring chloramines over bromamines was somewhat unexpected, because bromochloramine, which was predicted to form at the greatest extent during blended disinfection (Figure 4), was previously reported to form more NDMA than chloramines.^28,56^

Until recently, bromochloramine might have been expected to produce more NDMA than dichloramine, as the bromide in bromamines is a better leaving group in nucleophilic substitution reactions with DMBzA or other precursors,^55,76^ thought to lead to hydrazine intermediates toward NDMA formation.^29,44,55^ However, an update was recently proposed to the formation mechanism of NDMA from chloramines, in which the key step is hydrolysis of dichloramine, releasing reactive nitrogen species, which go on to react with amine precursors to form NDMA.^50^ Brominated species were not considered in this revised mechanism. Although similar nitrosating agents might be produced if bromochloramine and bromamines could analogously hydrolyze, the most recent assessment of bromamine formation and decomposition kinetics reported that no bromochloramine hydrolysis reaction was required for the model to fit experimental data.^58^ Therefore, bromochloramine hydrolysis may be too slow to account for the substantial production of nitrosating species.

Seawater disinfection prior to blending, which produced the least dichloramine and bromochloramine in the model, formed the smallest amount of NDMA in both synthetic and authentic water blending experiments (Figure 6). This is consistent with the literature reporting suppressed NDMA formation in the presence of bromide at neutral pH and low dichloramine concentrations.^55,56,77^ Decreased NDMA formation from seawater disinfection is potentially attributable to direct oxidation by free bromine of the amine functional group of DMBzA, which presumably competes for bromine with ammonia. Apparent rate constants at pH 7 of tertiary amine chlorination are approximately 2 orders of magnitude slower than ammonia chlorination,^78^ and analogously free bromination of ammonia is likely faster than tertiary amine bromination. However, the rate-limiting step in NDMA-forming reactions from bromamines might be slower than amine bromination by free bromine. Additionally, in the authentic blended water, the HOBr formed during seawater disinfection is likely consumed to some degree by reactions with natural organic matter in the seawater prior to blending.

### Effect of Blending Ratio on NDMA Formation during Disinfection

**Figure 7:**
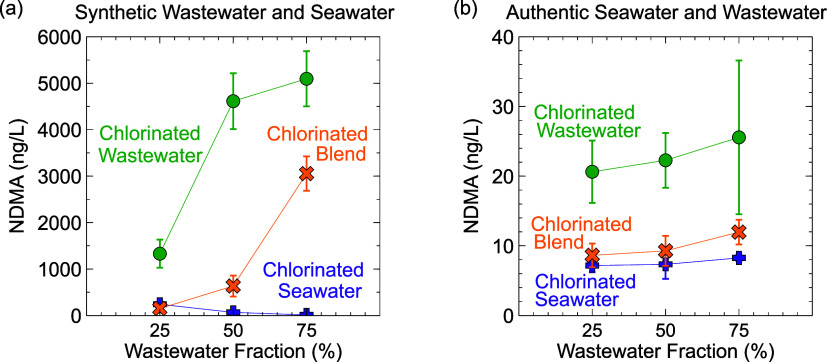
NDMA formation in (a) synthetic and (b) authentic seawater and wastewater as a function of wastewater fraction for seawater disinfection prior to blending (pink), wastewater disinfection prior to blending (green), and blended disinfection (in orange). Error bars represent standard deviations of three experimental replicates. Experimental conditions: pH = 7.0, [NaOCl]_0_ = 70.5 μM, [PO_4_]_tot_ = 10 mM, [DMBzA] = 50 μM in synthetic wastewater, *t*_rxn_ = 60 min, *T* = 22 ± 1 °C.

Analogous to the halamine speciation simulations, three blending ratios were disinfected experimentally at the bench scale (wastewater fraction = 25, 50, and 75%). During seawater disinfection, NDMA formation was low (Figure 7), and contrasting trends between synthetic and authentic waters were observed. In the synthetic water, NDMA formation decreased with increasing wastewater fraction (from 241 ± 19 ng/L at 25% wastewater to 12.1 ± 3 ng/L at 75% wastewater), while in the authentic waters, NDMA formation slightly increased with increasing wastewater fraction (from 7.2 ± 1.0 ng/L at 25% wastewater to 8.3 ± 0.5 ng/L, respectively) (Figure 7). The decline in NDMA production during synthetic seawater chlorination at higher wastewater fractions is consistent with modeling results showing a decline in dichloramine exposure and increase in monobromamine exposure in more wastewater-rich blends (Figure S5A). In authentic water, NDMA formation may have remained consistently low across all blending ratios during seawater chlorination, because the breakpoint was likely reached (Cl_TOT_/N_TOT_ = 1.48), and breakpoint bromination reactions are much faster than breakpoint chlorination (e.g., *k*_NH_2_Br,NHBr_2__ = 6.2 M^–1^ s^–1^ vs *k*_NH_2_Cl,NHCl_2__ = 0.015 M^–1^ s^–1^ (Table S1). During wastewater disinfection, in both the synthetic and authentic waters, NDMA formation increased with increasing wastewater fraction (i.e., increasing NDMA yield with increasing ammonia concentration and decreasing bromide concentration) (Figure 7). Blended disinfection followed a similar trend in which NDMA formation increased with an increasing wastewater fraction. In both cases, modeling predicted decreased dichloramine exposure at higher wastewater fractions (Figure 4), suggesting that dichloramine production alone cannot solely explain the rise in NDMA formation observed with increasing blending ratios. Therefore, we suspect that increased NDMA formation in more wastewater-rich blends was likely because of the presence of higher NDMA precursor concentrations and a higher relative proportion of chloramines to bromamines, as seen in the modeled halamine speciation discussed previously.

**Figure 8:**
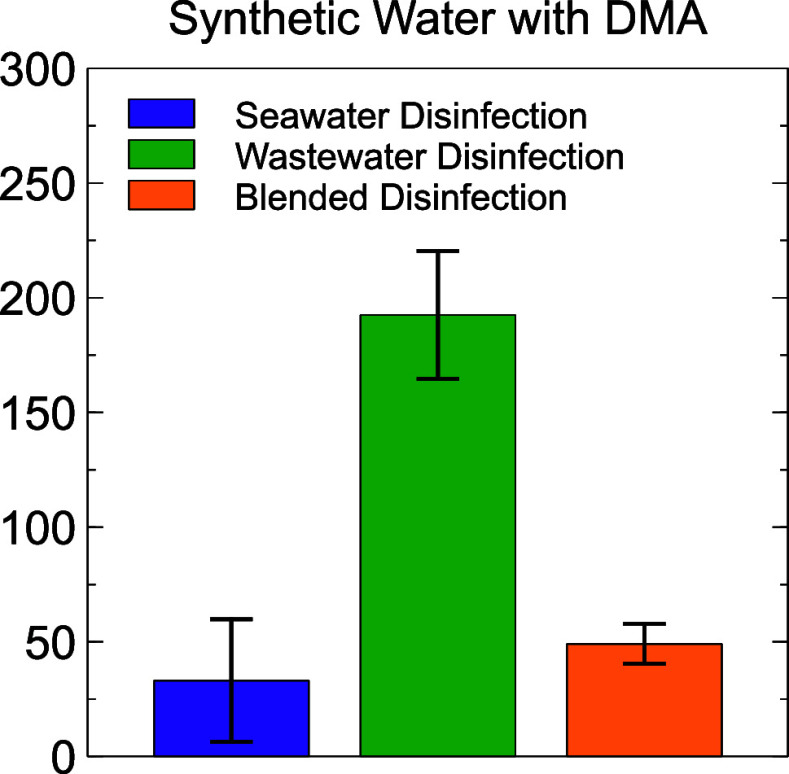
NDMA formation in synthetic waters with DMA as an NDMA precursor for the chlorinated seawater scenario (in purple), chlorinated wastewater scenario (in green), and chlorinated blend scenario (in orange), pH = 7.0, [NaOCl]_0_ = 70.5 μM, [PO_4_]_tot_ = 10 mM, [DMA]_0_ = 50 μM, *t*_rxn_ = 60 min, *T* = 22 ± 1 °C. Error bars represent standard deviations of three experimental replicates.

Additionally, the distribution of the NDMA yield between blending ratios was more pronounced in the synthetic waters than the authentic waters for all three blending scenarios. The dramatic shift in NDMA yield as a function of blending ratio in the synthetic waters was thought to be potentially attributable to the model NDMA precursor chosen, DMBzA, which is efficiently converted to NDMA, with molar yields approaching 100% under certain conditions.^41^ Thus, synthetic experiments were also conducted with DMA as the model precursor, which is less efficiently converted to NDMA, with typical molar yields of 3% or less.^29,40,55,79^ With DMA, NDMA yields were less strongly affected by the blending strategy than with DMBzA (Figure 8), and more closely resembled those observed when disinfecting authentic seawater and wastewater. This potentially suggests that the predominant NDMA precursors in authentic wastewater effluent more closely resemble low-yield precursors such as DMA rather than high-yield precursors such as DMBzA, ranitidine, and methadone, which have received extensive attention in the literature.^43,44,49,80^ A potentially simpler explanation for the contrasting trends between authentic and synthetic water disinfection is that breakpoint bromination was likely occurring in the authentic waters at 25 and 50% wastewater blending ratios, partially offsetting the lower abundance of NDMA precursors in less wastewater-rich blends with Cl/N molar ratios of 3.6:1 and 2.4:1, respectively. While breakpoint chlorination is known to promote NDMA,^81^ little is known about breakpoint bromination and should be studied further.

In both synthetic and authentic waters, NDMA was minimized in nearly all cases when first chlorinating seawater prior to blending. A seawater-rich blend was shown to further minimize NDMA formation in all scenarios with the exception of synthetic chlorinated seawater. In synthetic chlorinated seawater, NDMA formation was low at each blending ratio, and declined rather than increasing with increasing wastewater fraction (Figure 7A), consistent with declines in dichloramine exposure (Figure S5). The discrepancy between authentic and synthetic water NDMA formation when chlorinating seawater first may have arisen from the low ammonia concentration in the authentic wastewater (0.81 mg/L) compared to that in synthetic wastewater (10 mg/L), leading to breakpoint bromination in the authentic water after blending. This suggests that well- and poorly-nitrified wastewater effluents may respond differently to changes in blending ratio and that careful monitoring and possibly augmentation of background ammonia concentration may be necessary to avoid breakpoint chlorination and bromination.

## Conclusions

Halamine speciation modeling indicated that disinfecting wastewater prior to blending promoted dichloramine formation relative to bromochloramine compared to other scenarios. Bench-scale experiments using synthetic and authentic waters revealed that chlorinating wastewater prior to blending with seawater yielded the most NDMA formation while chlorinating seawater prior to blending with wastewater yielded the least. Moreover, NDMA formation increased as the wastewater fraction increased, suggesting that oxidant species formed during wastewater disinfection are principally responsible for NDMA formation. Elevated NDMA formation in water with higher dichloramine and lower bromochloramine formation was somewhat surprising because bromochloramine was previously found to promote NDMA formation in chloraminated waters containing bromide. Bromochloramine was predominantly formed when the stream was disinfected after blending; however, this scenario produced NDMA concentrations that were an order of magnitude lower than wastewater disinfection from model precursors and ∼2.5× times lower in authentic waters. This suggests that efforts to minimize NDMA formation should focus on pretreatment that minimizes dichloramine formation during disinfection rather than bromochloramine and that in a future wastewater–seawater blending scenario, seawater should be disinfected prior to blending with wastewater. Additionally, because NDMA “re-formation” has been observed at water reuse facilities in the final effluent after RO and UV/AOP^82^ and is affected by RO-induced shifts in chloramine speciation,^74^ halamine speciation during advanced treatment of blended waters may warrant further consideration. In each scenario, the high levels of bromide and iodine in seawater are likely to promote brominated and iodinated DBP formation, which may constrain disinfection options.

## Supporting Information Available

Supporting Information reaction rate constants, representative concentrations of relevant species in seawater and wastewater, water quality parameters, halamine kinetics model validation data, modeled halamine concentrations and exposures (PDF)
