# Supplementary material for: Minimizing N-Nitrosodimethylamine Formation During Disinfection of Blended Seawater and Wastewater Effluent
Source: ACS ES T Water. 2024 Feb 6;4(4):1498–507. doi: 10.1021/acsestwater.3c00617 (PMC11019544; doi:10.1021/acsestwater.3c00617)
Supplement: Supplementary file 1 — ew3c00617_si_001.pdf [file ew3c00617_si_001.pdf]

Supporting Information for

Minimizing *N*-nitrosodimethylamine Formation during Disinfection of Blended Seawater and Wastewater Effluent

Sophia L. Plata[1,2], Amy E. Childress[1], Daniel L. McCurry[1,]*

[1] Astani Department of Civil and Environmental Engineering, University of Southern California, Los Angeles, CA 90089

[2] Current Affiliation: Department of Engineering, Harvey Mudd College, Claremont, CA 91711

*Corresponding author phone: (213) 740-0762; e-mail: dmccurry@usc.edu

20 pages

6 Tables

6 Figures

Contents

Text S1: NDMA extraction, quantification, and detection limit determination…………………S3

Table S1: Chemical equations and rate constants applied to Kintecus model…………..…. S4-S5

Table S2: Typical bromide, chlorine, ammonia, and chloride concentrations of secondary treated wastewater and seawater……………………………………………………………………………S6

Table S3: Typical ionic strength values of secondary treated wastewater and seawater…………S7

Table S4: Typical pH values of secondary treated wastewater and seawater……………………..S7

Table S5: Composition of secondary treated wastewater and seawater used in bench-scale disinfection experiments……………………………………………………………………S8

Table S6: QA/QC data to determine detection limit of NDMA using EPA Method 552.3………S8

Figure S1: Model comparison of (a) Jafvert and Valentine 2012 and (b) this work………………S9

Figure S2: Model comparison of (a) Luh and Mariñas 2014 and (b) this work…………………...S9

Figure S3. Chloramine and bromamine species concentrations over time modeled as a function of wastewater fraction for the (a) chlorinated seawater scenario, (b) chlorinated wastewater scenario, and (c) chlorinated blend scenario. …………………………………………………………..….S10

Figure S4: Cumulative chloramine and bromamine exposures modeled as a function of blending approach for (a) chlorinated seawater scenario, (b) chlorinated wastewater scenario, and (c) chlorinated blend. ……………..……………………………………………………..….S10

Figure S5: Cumulative halamine exposures modeled as a function of wastewater fraction for (a) seawater disinfection prior to blending, (b) wastewater disinfection prior to blending, and (c) blended disinfection………………………………………………………………………S11

Figure S6: Example NDMA calibration curves (A) extracted from aqueous standards, and (B) prepared directly in dichloromethane……………………………………………………..S11

Text S1. NDMA extraction, quantification, and detection limit determination

Extraction and quantification. NDMA analytical standards were prepared by spiking a range of NDMA concentrations from a 5 mg/L stock solution (prepared in dichloromethane) into 23.5 mL of 10 mM pH 7 phosphate buffer prepared in Milli-Q water in a 23.5 mL borosilicate vials capped with PTFE-faced septa. Standards were spiked with 10 µg/L of d6-NDMA (as a mass-labeled surrogate) from a 2 mg/L stock (prepared in acetonitrile). Standards were extracted in the same manner as experimental samples: 23.5 mL samples were transferred to 40 mL borosilicate vials containing 10% v/v (2.35 mL) of dichloromethane, capped with PTFE-faced septa, and shaken for two minutes. After phase separation, the dichloromethane phase was removed, dried with magnesium sulfate heptahydrate, and transferred to 2 mL autosampler vials for GC/MS/MS analysis. An example extracted calibration curve is provided in **Figure S6A**.

Extraction efficiency. Extraction efficiency was calculated as the volume-normalized average peak area ratio of d6-NDMA in six extracted standards compared to six standards prepared at the same concentration in dichloromethane (**Figure S6B**):

$$Extraction\ effciency = \frac{d6\ area_{extracted}}{d6\ area_{solvent}}\left(\frac{Volume_{solvent}}{Volume_{extracted}}\right) = \frac{21912}{6297}\left(\frac{2.35\ mL}{23.5\ mL}\right) = 35\%$$

Limit of detection. The method detection limit was determined by preparing seven replicate 23.5 mL standards in 10 mM phosphate buffer (pH 7) at a concentration estimated to produce a signal-to-noise ratio of 5:1, and extracting and analyzing in the same manner as experimental samples. The standard deviation of the seven concentrations was multiplied by the Student's t-test value corresponding to a 99% confidence interval (3.143) to produce the detection limit (2.8 ng/L) (**Table S6**) [1].

Table S1. Equations and rate constants used to model disinfectant speciation in Kintecus

**Chloramines**	**Rate Constant (M^{-1} s^{-1})**	**Reference**
HOCl + NH_3 ==> NH_2Cl	4.20×10^{6}	[2]
NH_2Cl ==> HOCl + NH_3	2.11×10^{-5}	[2]
HOCl + NH_2Cl ==> $NHCl_2$	2.78×10^{2}	[2]
$NHCl_2$ ==> HOCl + NH_2Cl	6.50×10^{-7}	[2]
NH_2Cl + NH_2Cl ==> $NHCl_2$ + NH_3	$(6.90 \times 10^{3})[H^{+}]$ + 899 $[H_3PO_4]$ + $0.36[H_2PO_4^{-}]$	[2]
$NHCl_2$ + NH_3 ==> NH_2Cl + NH_2Cl	6.00×10^{4} $[H^{+}]$	[2]
$NHCl_2$ ==> I	1.11×10^{2} $[OH^{-}]$	[2]
I + $NHCl_2$ ==> HOCl + prod	2.78×10^{4}	[2]
I + NH_2Cl ==> prod1	8.33×10^{3}	[2]
$NHCl_2$ + NH_2Cl ==> prod2	1.53×10^{-2}	[2]
HOCl + $NHCl_2$ ==> NCl_3	$(1.60 \times 10^{4})[HPO_4^{2-}] + (9.00 \times 10^{4})[OCl^{-}]$ + $(3.30 \times 10^{9})[OH^{-}]$	[2]
NCl_3 ==> HOCl + $NHCl_2$	3.20×10^{-5} $(1 + 5.88 \times 10^{5}$ $[OH^{-}])$	[2]
$NHCl_2$ + NCl_3 ==> HOCl + HOCl + prod4	$(5.60 \times 10^{10})[OH^{-}]$	[2]
NH_2Cl + NCl_3 ==> HOCl + prod5	$(1.40 \times 10^{9})[OH^{-}]$	[2]
$NHCl_2$ + OCl^{-} ==> NO_3^{-} + $4Cl^{-}$	2.31×10^{2}	[2]
NH_2Cl + Br^{-} ==> NH_3 + BrCl	$(3.80 \times 10^{5})[H^{+}]$	[3]
**Free Bromine**	**Rate Constant (M^{-1} s^{-1})**	
BrCl + Br^{-} ==> Br_2 + Cl^{-}	1.00×10^{8}	[4], [5]
BrCl ==> HOBr + Cl^{-}	$(3.00 \times 10^{6}) + 10^{9.8}[OH^{-}]$	[4], [5]
HOBr + Cl^{-} ==> BrCl	$(2.30 \times 10^{10})[H^{+}]$	[4], [5]
Br_2 ==> HOBr + Br^{-}	$97 + 10^{9.8}[OH^{-}]$	[6]
HOBr + Br^{-} ==> Br_2	$(1.60 \times 10^{10})[H^{+}]$	[6]
HOCl + Br^{-} ==> BrCl	1.32×10^{6} $[H^{+}]$	[7]
HOCl + Br^{-} ==> HOBr	1.55×10^{3}	[7]
OCl^{-} + Br^{-} ==> OBr^{-} + Cl^{-}	9.00×10^{-4}	[7]
**Bromamines**	**Rate Constant (M^{-1} s^{-1})**	
HOBr + NH_3 ==> NH_2Br	7.50×10^{7}	[8]
NH_2Br + NH_2Br ==> $NHBr_2$ + NH_3	$0.5 + (5.00 \times 10^{8})[H^{+}] + 290[NH_4^{+}]$ + $(3.40 \times 10^{4})[H_2PO_4^{-}]$	[9]
$NHBr_2$ + NH_3 ==> NH_2Br + NH_2Br	$1 + (1.00 \times 10^{9})[H^{+}] + (6.50 \times 10^{4})[H_2PO_4^{-}]$	[9]
NH_2Br + $NHBr_2$ ==> prod6 + $3Br^{-}$	$6.20 + 8.30 \times 10^{4}$ $[OH^{-}]$	[9]
$NHBr_2$ + $NHBr_2$ ==> prod7 + HOBr + $3Br^{-}$	$8.90 + 1.40 \times 10^{4}$ $[HPO_4^{2-}]$	[9]
NH_2Br + Br^{-} ==> NH_3 + Br_2	3.60×10^{9} $[OH^{-}]$	[3]
NH_2Br + Br_2 ==> $NHBr_2$ + Br^{-}	4.60×10^{6}	[3]
**Bromochloramine formation**	**Rate Constant (M^{-1} s^{-1})**	
NH_2Cl + Br_2 ==> NHBrCl + Br^{-}	4.18×10^{8}	[10]
NH_2Cl + Br_3^{-} ==> NHBrCl + $2Br^{-}$	3.07×10^{7}	[10]
NH_2Cl + HOBr ==> NHBrCl	2.86×10^{5}	[10]
NH_2Cl + OBr^{-} ==> NHBrCl	2.20×10^{4}	[10]
NH_2Cl + NH_2Br ==> NHBrCl + NH_3	$(3.10 \times 10^{7})[H^{+}]$	[3]

$NH_2Cl + NHBr_2$ ==> $NHBrCl$ + prod8 + Br^-	1.90×10^{-1}	[3]
**Bromochloramine decomposition**	**Rate Constant ($M^{-1}\ s^{-1}$)**	
$NHBrCl + NHBrCl$ ==> N_2 + 2Clm + Br^- + OBr^-	$0.52 + (1.50 \times 10^{-3})[OH^-] + 139\ [HPO_4^{2-}]$	[3]
$NHBrCl + NH_2Cl$ ==> N_2 + 2Clm + Br^-	5.60×10^{-6}	[3]
$NHBrCl + NH_2Br$ ==> prod9 + $2Br^-$	7.10×10^{-2}	[3]
$NHBrCl + NHBr_2$ ==> prod10 + $HOBr$ + $2Br^-$	$(1.40 \times 10^{3}) + 41.7[OH^-] + 1389\ [HPO_4^{2-}]$	[3]
$NHBrCl + NH_3$ ==> $NH_2Cl + NH_2Br$	$3.90 \times 10^{9}\ [H^+]$	[3]
$NHBrCl + Br^-$ ==> $NH_2Cl + Br_2$	$3.30 \times 10^{9}\ [H^+]$	[3]
**Acid-Base Reactions**	**Rate Constant ($M^{-1}\ s^{-1}$)**	
$HOCl$ ==> OCl^-	3.16×10^{2}	[11]
OCl^- ==> $HOCl$	1.00×10^{3}	[11]
NH_4^+ ==> NH_3	5.62×10^{0}	[12]
NH_3 ==> NH_4^+	1.00×10^{3}	[12]
DMA^+ ==> DMA	2.00×10^{-1}	[13]
DMA ==> DMAp	1.00×10^{3}	[13]
UDMHp ==> UDMH	6.31×10^{2}	[13]
UDMH ==> $UDMH^+$	1.00×10^{3}	[13]
$HOBr$ ==> OBr^-	1.59×10^{3}	[12]
OBr^- ==> $HOBr$	1.00×10^{5}	[12]
$Br_2 + Br^-$ ==> Br_3^-	7.94×10^{-5}	[12]
Br_3^- ==> $Br_2 + Br^-$	1.00×10^{12}	[12]
**DMA Chlorination**	**Rate Constant ($M^{-1}\ s^{-1}$)**	
DMA + $HOCl$ ==> DMACl	6.05×10^{7}	[13]
$DMA^+ + NH_2Cl$ ==> DMACl + NH_4^+	2.10×10^{-1}	[13]
DMACl + NH_4^+ ==> $NH_2Cl + DMA^+$	5.80×10^{-3}	[13]
**NDMA Formation**	**Rate Constant ($M^{-1}\ s^{-1}$)**	
NH_2Cl + DMA ==> UDMH	8.10×10^{-2}	[13]
NH_3 + DMACl ==> UDMH	4.90×10^{-3}	[13]
$NHCl_2$ + DMA ==> UDMHCl	5.20×10^{1}	[13]
$NHCl_2$ + UDMH ==> DMA + PRODUCTS	4.50×10^{0}	[13]
$NHCl_2$ + UDMHCl ==> PRODUCTS	7.50×10^{-1}	[13]
UDMHCl + O_2 ==> NDMA	1.40×10^{0}	[13]
DMACl ==> $CH_3NCH_2 + Cl^-$	4.20×10^{-12}	[13]

Table S2: Typical bromide, chlorine, ammonia, and chloride concentrations of secondary treated wastewater and seawater. Concentrations used in "This study" refers to the value used for modeling experiments and synthetic experiments.

Constituent	Wastewater (mg/L)	Ref	Seawater (mg/L)	Ref
Bromide (Br^-)	< 0.1	[14]	63	[15]
	1.4	[16]	60 – 78	[17]
	0.52	[18]		
	1.03	[19]		
	0.22	[20]		
	0.14	[21]		
	0.05	[21]		
	0.025 – 0.151	[22]		
	0.1	This study	63	This study
Chloride (Cl^-)	141	[23]	19,400	[24]
	309	[18]	18,980	[25]
	66.5	[26]	19,162	[27]
	70.9	[26]	18,980	[28]
	514.6	[26]		
	187.8	[26]		
	52.2	[29]		
	326	[19]		
	165	This study	19,000	This study
Ammonia (NH_3)	10	[30]	0.01	[31]
	5.4	[21]		
	1	[21]		
	62	[32]		
	10	[33]		
	11.5	[34]		
	7.6	[35]		
	0.3	[36]		
	0.4	[36]		
	10	This study	0.01	This study
Cl_2 Dose	5 of monochloramine (1 – 2 target residual)	[37]	0.5 – 2.0	[38], [39]
	3 - 4	[40]	3	[41]
	2 - 4	[42]	< 1	[43]
	5	This study	1	This study

Table S3: Typical ionic strength values of secondary treated wastewater and seawater. "This study" refers to the value used for synthetic water experiments.

Stream	μ (M)	Notes	Ref
**Secondary Wastewater Effluent**	0.05	Used to simulate 2° trt WW experiments	[44]
	0.0147	Used to simulate 2° trt WW experiments	[45]
	0.0035	Characterized from Japanese WWTP	[46]
	0.01	This study	
**Seawater**	0.7	Used to simulate SW experiments	[47]
	0.7	Used to simulate SW experiments	[48]
	0.7	Ppr on physical chemistry of SW	[49]
	0.7231	Artificial SW ionic strength	[50]
	0.70	This study	

Table S4: Typical pH values of secondary treated wastewater and seawater. "This study" refers to the value used for synthetic water experiments.

Stream	pH	Notes	Ref
**Secondary Wastewater Effluent**	7.71 ± 0.09	Wilmington WWTP in DE	[44]
	7.29	WWTP in Easton, PA	[51]
	8.0 ± 0.2	WWTP in Mostoles, Madrid, Spain	[52]
	7.4	Used to simulate 2° treatment WW experiments	[45]
	6.8	Japanese WWTP	[46]
	7.31	2° effluent collected from local water reclamation plant (Singapore)	[53]
	7.0 ± 0.1	Gyeongsan City WWTP in South Korea	[54]
	7.0 ± 0.2	Municipal WWTP in Queensland, Australia	[18]
	7.95	Nine Springs WWTP in Madison, WI	[55]
	7.50	This study	
**Seawater**	8.1	From textbook: Physical Chemistry of Seawater	[49]
	8.136 ± 0.003	Measurements taken from Gulf of Maine	[56]
	7.4 – 8.4	Used to simulate SW	[57]
	7.4 – 8.4	Computed based on literature	[58]
	8.10	This study	

Table S5. Composition of secondary treated wastewater and seawater used in bench-scale disinfection experiments.

Constituent	Wastewater	Seawater	Unit
pH	7.13	7.93	-
Cl^-	260	19,360	mg/L
Br^-	0.07	64.5	mg/L
NO_2^-	0.87	<0.05	mg/L
NO_3^-	7.68	<1.00	mg/L
NH_3	0.81	<0.50	mg/L
TOC	11.1	1.25	mg/L

Table S6. NDMA limit of detection determination.

	Area counts		Area ratio	[NDMA]
Replicate	NDMA	d6-NDMA	NDMA/d6	(ng/L)
1	182.5	120460	0.00152	7.4
2	193	112979	0.00171	8.3
3	116	82965	0.00140	6.8
4	156	91349	0.00171	8.3
5	225	130097	0.00173	8.4
6	527	272665	0.00193	9.4
7	473	258760	0.00183	8.9
			average	8.2
			std. dev.	0.9
			**LOD**	**2.8**

Figure S1: Model comparison of (a) Jafvert and Valentine 1992 [59] and (b) this work.

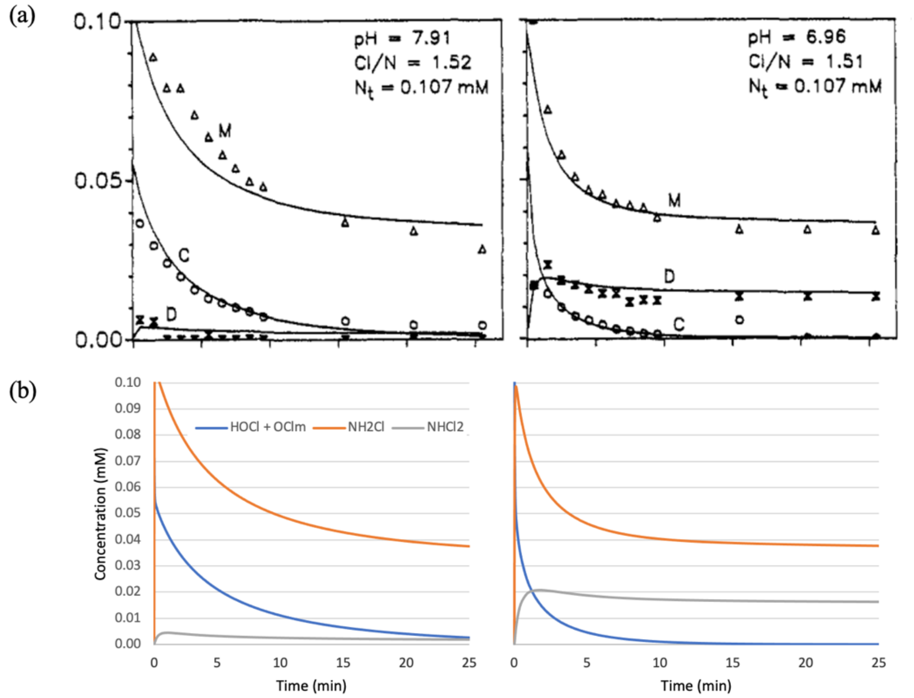

Figure S2: Model comparison of (a) Luh and Mariñas 2014 [3] and (b) this work.

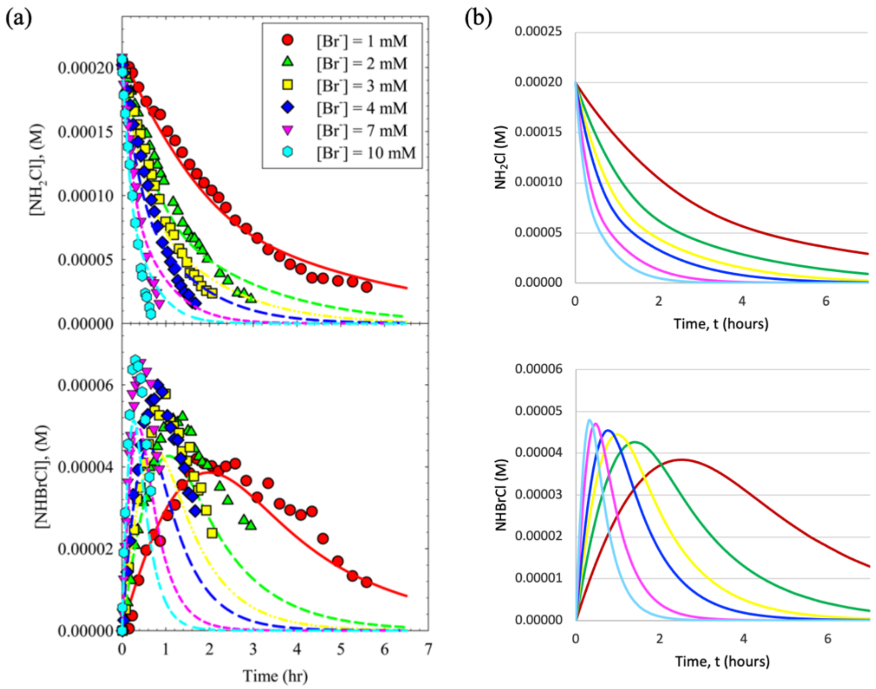

Figure S3. Chloramine and bromamine species concentrations over time modeled as a function of wastewater fraction for the (a) chlorinated seawater scenario, (b) chlorinated wastewater scenario, and (c) chlorinated blend scenario.

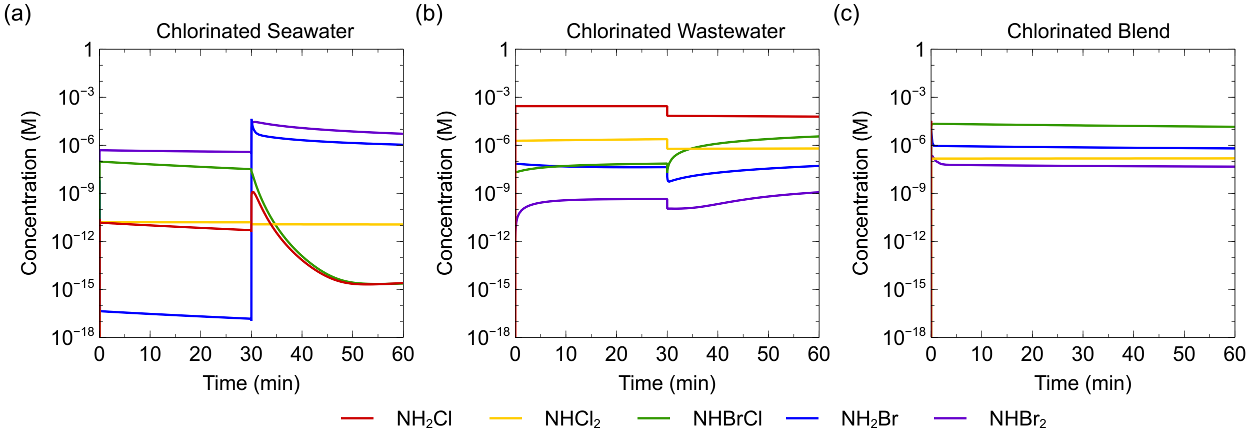

Figure S4. Cumulative chloramine and bromamine exposures modeled as a function of blending approach for (a) chlorinated seawater scenario, (b) chlorinated wastewater scenario, and (c) chlorinated blend.

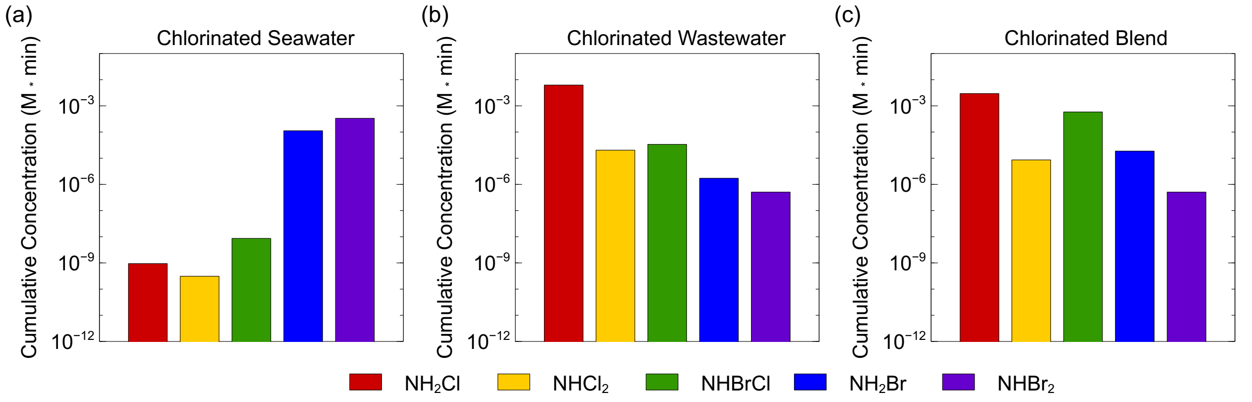

Figure S5. Cumulative halamine exposures modeled as a function of wastewater fraction for (a) seawater disinfection prior to blending, (b) wastewater disinfection prior to blending, and (c) blended disinfection.

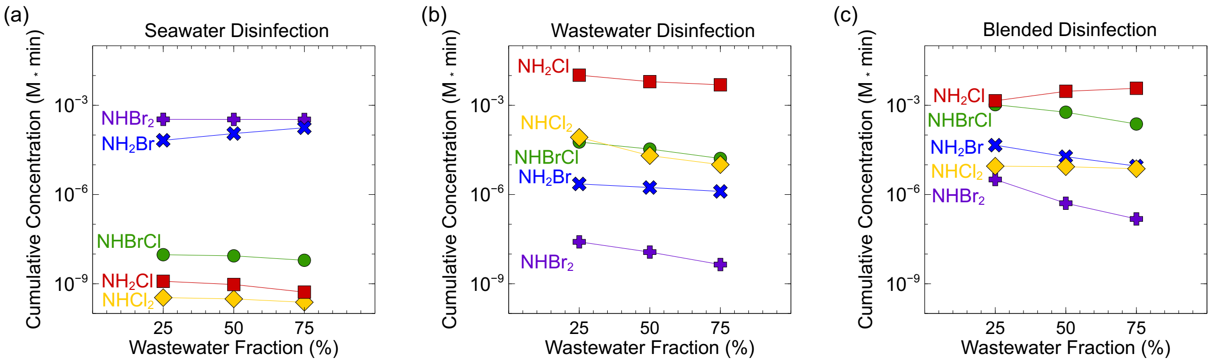

Figure S6. Example NDMA calibration curves (A) extracted from aqueous standards, and (B) prepared directly in dichloromethane

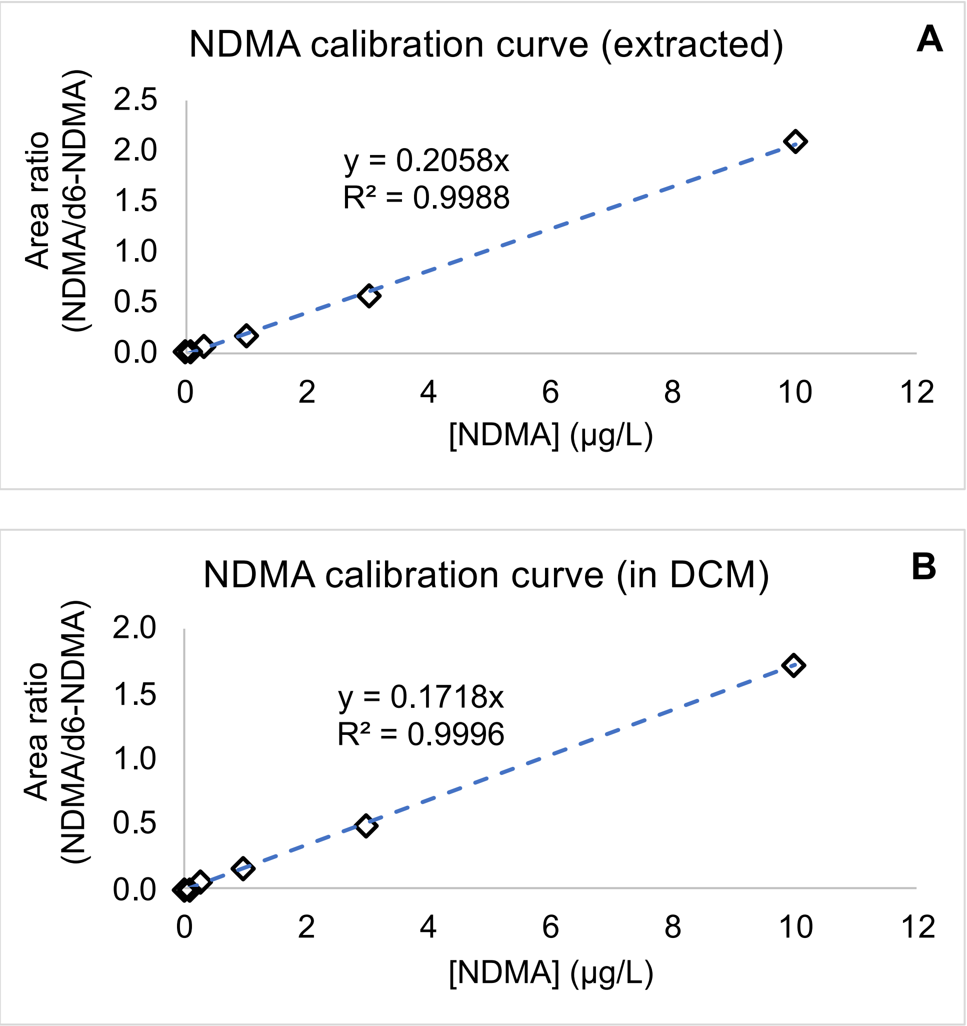

References

[1] M. M. Domino, B. V Pepich, D. J. Munch, P. S. Fair, and Y. Xie, “Method 552.3 determination of haloacetic acids and dalapon in drinking water by liquid-liquid microextraction, derivatization, and gas chromatography with electron capture detection,” *Environ. Prot. Agency, Cincinnati, OH*, 2003.

[2] C. T. Jafvert and R. L. Valentine, “Reaction scheme for the chlorination of ammoniacal water,” *Environ. Sci. Technol.*, vol. 26, no. 3, pp. 577–586, Mar. 1992, doi: 10.1021/es00027a022.

[3] J. Luh and B. J. Mariñas, “Kinetics of Bromochloramine Formation and Decomposition,” *Environ. Sci. Technol.*, vol. 48, no. 5, pp. 2843–2852, Mar. 2014, doi: 10.1021/es4036754.

[4] T. X. Wang, M. D. Kelley, J. N. Cooper, R. C. Beckwith, and D. W. Margerum, “Equilibrium, Kinetic, and UV-Spectral Characteristics of Aqueous Bromine Chloride, Bromine, and Chlorine Species,” *Inorg. Chem.*, vol. 33, no. 25, pp. 5872–5878, Dec. 1994, doi: 10.1021/ic00103a040.

[5] Q. Liu and D. W. Margerum, “Equilibrium and Kinetics of Bromine Chloride Hydrolysis,” *Environ. Sci. Technol.*, vol. 35, no. 6, pp. 1127–1133, Mar. 2001, doi: 10.1021/es001380r.

[6] R. C. Beckwith, T. X. Wang, and D. W. Margerum, “Equilibrium and Kinetics of Bromine Hydrolysis,” *Inorg. Chem.*, vol. 35, no. 4, pp. 995–1000, Jan. 1996, doi: 10.1021/ic950909w.

[7] K. Kumar and D. W. Margerum, “Kinetics and mechanism of general-acid-assisted oxidation of bromide by hypochlorite and hypochlorous acid,” *Inorg. Chem.*, vol. 26, no. 16, pp. 2706–2711, Aug. 1987, doi: 10.1021/ic00263a030.

[8] J. E. Wajon and J. C. Morris, “Rates of formation of N-bromo amines in aqueous solution,” *Inorg. Chem.*, vol. 21, no. 12, pp. 4258–4263, 1982.

[9] H. Lei, B. J. Mariñas, and R. A. Minear, “Bromamine Decomposition Kinetics in Aqueous Solutions,” *Environ. Sci. Technol.*, vol. 38, no. 7, pp. 2111–2119, Apr. 2004, doi: 10.1021/es034726h.

[10] M. Gazda and D. W. Margerum, “Reactions of Monochloramine with Br2, Br3-, HOBr, and OBr-: Formation of Bromochloramines,” *Inorg. Chem.*, vol. 33, pp. 118–123, 1994.

[11] L. C. Adam, I. Fabian, K. Suzuki, and G. Gordon, “Hypochlorous acid decomposition in the pH 5-8 region,” *Inorg. Chem.*, vol. 31, no. 17, pp. 3534–3541, Aug. 1992, doi: 10.1021/ic00043a011.

[12] R. Smith and A. Constants, “Vol. 4: Inorganic Complexes.” Plenum Press, New York and London, 1976.

[13] M. E. Huang, S. Huang, and D. L. McCurry, “Re-Examining the Role of Dichloramine in High-Yield N-Nitrosodimethylamine Formation from N,N-Dimethyl-α-arylamines,” *Environ. Sci. Technol. Lett.*, vol. 5, no. 3, pp. 154–159, Mar. 2018, doi: 10.1021/acs.estlett.7b00572.

[14] Q.-Y. Wu, Y. Li, H.-Y. Hu, Y.-X. Sun, and F.-Y. Zhao, “Reduced Effect of Bromide on the Genotoxicity in Secondary Effluent of a Municipal Wastewater Treatment Plant during Chlorination,” *Environ. Sci. Technol.*, vol. 44, no. 13, pp. 4924–4929, Jul. 2010, doi: 10.1021/es100152j.

[15] A. Suzuki, L. W. Lim, T. Hiroi, and T. Takeuchi, “Rapid determination of bromide in seawater samples by capillary ion chromatography using monolithic silica columns modified with cetyltrimethylammonium ion,” *Talanta*, vol. 70, no. 1, pp. 190–193, 2006,

doi: https://doi.org/10.1016/j.talanta.2005.12.064.

[16] Y.-X. Sun, Q.-Y. Wu, H.-Y. Hu, and J. Tian, “Effect of bromide on the formation of disinfection by-products during wastewater chlorination,” *Water Res.*, vol. 43, no. 9, pp. 2391–2398, 2009, doi: https://doi.org/10.1016/j.watres.2009.02.033.

[17] P. Dorji, J. Choi, D. I. Kim, S. Phuntsho, S. Hong, and H. K. Shon, “Membrane capacitive deionisation as an alternative to the 2nd pass for seawater reverse osmosis desalination plant for bromide removal,” *Desalination*, vol. 433, pp. 113–119, 2018, doi: https://doi.org/10.1016/j.desal.2018.01.020.

[18] S. Garcia-Segura, J. Keller, E. Brillas, and J. Radjenovic, “Removal of organic contaminants from secondary effluent by anodic oxidation with a boron-doped diamond anode as tertiary treatment,” *J. Hazard. Mater.*, vol. 283, pp. 551–557, 2015, doi: https://doi.org/10.1016/j.jhazmat.2014.10.003.

[19] B. Ruffino, G. V Korshin, and M. Zanetti, “Use of spectroscopic indicators for the monitoring of bromate generation in ozonated wastewater containing variable concentrations of bromide,” *Water Res.*, vol. 182, p. 116009, 2020, doi: https://doi.org/10.1016/j.watres.2020.116009.

[20] Y. Zhong, W. Gan, Y. Du, H. Huang, Q. Wu, Y. Xiang, C. Shang, and X. Yang, “Disinfection byproducts and their toxicity in wastewater effluents treated by the mixing oxidant of ClO2/Cl2,” *Water Res.*, vol. 162, pp. 471–481, 2019, doi: https://doi.org/10.1016/j.watres.2019.07.012.

[21] Y.-X. Sun, Q.-Y. Wu, H.-Y. Hu, and J. Tian, “Effect of ammonia on the formation of THMs and HAAs in secondary effluent chlorination,” *Chemosphere*, vol. 76, no. 5, pp. 631–637, 2009, doi: https://doi.org/10.1016/j.chemosphere.2009.04.041.

[22] H. Song, J. W. Addison, J. Hu, and T. Karanfil, “Halonitromethanes formation in wastewater treatment plant effluents,” *Chemosphere*, vol. 79, no. 2, pp. 174–179, 2010, doi: 10.1016/j.chemosphere.2010.01.001.

[23] W. R. Kelly, S. V Panno, K. C. Hackley, H.-H. Hwang, A. T. Martinsek, and M. Markus, “Using chloride and other ions to trace sewage and road salt in the Illinois Waterway,” *Appl. Geochemistry*, vol. 25, no. 5, pp. 661–673, 2010, doi: https://doi.org/10.1016/j.apgeochem.2010.01.020.

[24] “Chloride and Salinity,” *Columbia University*, 2011. https://www.ldeo.columbia.edu/edu/k12/snapshotday/activities/2011/Classroom HS activity/chloride conversion/Chloride and Salinity.pdf

[25] “Composition of seawater,” *Lenntech*, 2005. https://www.lenntech.com/composition-seawater.htm

[26] J. Choi, H. Lee, Y. Choi, S. Kim, S. Lee, S. Lee, W. Choi, and J. Lee, “Heterogeneous photocatalytic treatment of pharmaceutical micropollutants: Effects of wastewater effluent matrix and catalyst modifications,” *Appl. Catal. B Environ.*, vol. 147, pp. 8–16, 2014, doi: https://doi.org/10.1016/j.apcatb.2013.08.032.

[27] R. H. Bryne, A. C. Duxbury, and F. T. Mackenzie, “Seawater,” *Encyclopedia Britannica*. 2020. [Online]. Available: https://www.britannica.com/science/seawater

[28] “Mineral Makeup of Seawater,” *Stanford University*. https://web.stanford.edu/group/Urchin/mineral.html

[29] N. Dunkin, S. Weng, C. G. Coulter, J. G. Jacangelo, and K. J. Schwab, “Impacts of virus processing on human norovirus GI and GII persistence during disinfection of municipal secondary wastewater effluent,” *Water Res.*, vol. 134, pp. 1–12, 2018, doi:

https://doi.org/10.1016/j.watres.2018.01.053.

[30] S. Cho, T. T. Luong, D. Lee, Y.-K. Oh, and T. Lee, "Reuse of effluent water from a municipal wastewater treatment plant in microalgae cultivation for biofuel production," *Bioresour. Technol.*, vol. 102, no. 18, pp. 8639–8645, 2011, doi: https://doi.org/10.1016/j.biortech.2011.03.037.

[31] M. I. Liddicoat, S. Tibhitts, and E. I. Butler, "The determination of ammonia in seawater," *Limnol. Oceanogr.*, vol. 20, no. 1, pp. 131–132, Jan. 1975, doi: 10.4319/lo.1975.20.1.0131.

[32] A. Ashkanani, F. Almomani, M. Khraisheh, R. Bhosale, M. Tawalbeh, and K. AlJaml, "Bio-carrier and operating temperature effect on ammonia removal from secondary wastewater effluents using moving bed biofilm reactor (MBBR)," *Sci. Total Environ.*, vol. 693, p. 133425, 2019, doi: https://doi.org/10.1016/j.scitotenv.2019.07.231.

[33] B. Wang and C. Q. Lan, "Biomass production and nitrogen and phosphorus removal by the green alga Neochloris oleoabundans in simulated wastewater and secondary municipal wastewater effluent," *Bioresour. Technol.*, vol. 102, no. 10, pp. 5639–5644, 2011, doi: https://doi.org/10.1016/j.biortech.2011.02.054.

[34] B. W. Mercer, L. L. Ames, C. J. Touhill, W. J. Van Slyke, and R. B. Dean, "Ammonia Removal from Secondary Effluents by Selective Ion Exchange," *J. (Water Pollut. Control Fed.*, vol. 42, no. 2, pp. R95–R107, Jan. 1970, [Online]. Available: http://www.jstor.org.libproxy1.usc.edu/stable/25036497

[35] J. Kim, B. P. Lingaraju, R. Rheaume, J. Lee, and K. F. Siddiqui, "Removal of ammonia from wastewater effluent by chlorella vulgaris," *Tsinghua Sci. Technol.*, vol. 15, no. 4, pp. 391–396, 2010, doi: 10.1016/S1007-0214(10)70078-X.

[36] Q.-Y. Wu, H.-Y. Hu, X. Zhao, and Y. Li, "Effects of chlorination on the properties of dissolved organic matter and its genotoxicity in secondary sewage effluent under two different ammonium concentrations," *Chemosphere*, vol. 80, no. 8, pp. 941–946, 2010, doi: https://doi.org/10.1016/j.chemosphere.2010.05.005.

[37] D. L. McCurry, S. W. Krasner, and W. A. Mitch, "Control of nitrosamine during non-potable and de facto wastewater reuse with medium pressure ultraviolet light and preformed monochloramine," *Environ. Sci. Water Res. Technol.*, vol. 2, pp. 502–510, 2016.

[38] D. Kim, G. L. Amy, and T. Karanfil, "Disinfection by-product formation during seawater desalination: A review," *Water Res.*, vol. 81, pp. 343–355, 2015, doi: https://doi.org/10.1016/j.watres.2015.05.040.

[39] E. Agus, N. Voutchkov, and D. L. Sedlak, "Disinfection by-products and their potential impact on the quality of water produced by desalination systems: A literature review," *Desalination*, vol. 237, no. 1, pp. 214–237, 2009, doi: https://doi.org/10.1016/j.desal.2007.11.059.

[40] M. Sgroi, P. Roccaro, G. L. Oelker, and S. A. Snyder, "N-nitrosodimethylamine (NDMA) formation at an indirect potable reuse facility," *Water Res.*, vol. 70, pp. 174–183, 2015, doi: https://doi.org/10.1016/j.watres.2014.11.051.

[41] (Lenntech), "Desalination Pretreatment: Seawater chlorination," 2019. https://www.lenntech.com/processes/desalination/pretreatment/pretreatment/seawater-chlorination.htm

[42] S. E. Miller, R. A. Rodriguez, and K. L. Nelson, "Removal and growth of microorganisms across treatment and simulated distribution at a pilot-scale direct potable reuse facility,"

Environ. Sci. Water Res. Technol., vol. 6, pp. 1370–1387, 2020, [Online]. Available: https://pubs-rsc-org.libproxy1.usc.edu/en/content/articlehtml/2020/ew/c9ew01087d

[43] E. Agus and D. L. Sedlak, "Formation and fate of chlorination by-products in reverse osmosis desalination systems," *Water Res.*, vol. 44, no. 5, pp. 1616–1626, 2010, doi: https://doi.org/10.1016/j.watres.2009.11.015.

[44] C. P. Huang, P. Chiu, S. P. Myoda, I.-K. Kim, and M. Sung, "Treatment of Wastewaters for Water Reuse by a Catalytic Sonochemical Process," Denver, CO, 2011. [Online]. Available: https://www.usbr.gov/research/dwpr/reportpdfs/report48.pdf

[45] M. Herzberg, T. Z. Rezene, C. Ziemba, O. Gillor, and K. Mathee, "Impact of Higher Alginate Expression on Deposition of Pseudomonas aeruginosa in Radial Stagnation Point Flow and Reverse Osmosis Systems," *Environ. Sci. Technol.*, vol. 43, no. 19, pp. 7376–7383, Oct. 2009, doi: 10.1021/es901095u.

[46] Y. Yang, N. Nakada, R. Nakajima, M. Yasojima, C. Wang, and H. Tanaka, "pH, ionic strength and dissolved organic matter alter aggregation of fullerene C60 nanoparticles suspensions in wastewater," *J. Hazard. Mater.*, vol. 244–245, pp. 582–587, 2013, doi: https://doi.org/10.1016/j.jhazmat.2012.10.056.

[47] E. A. Christenson and J. Schijf, "Stability of YREE complexes with the trihydroxamate siderophore desferrioxamine B at seawater ionic strength," *Geochim. Cosmochim. Acta*, vol. 75, no. 22, pp. 7047–7062, 2011, doi: https://doi.org/10.1016/j.gca.2011.09.022.

[48] J. Schijf, E. A. Christenson, and K. J. Potter, "Different binding modes of Cu and Pb vs. Cd, Ni, and Zn with the trihydroxamate siderophore desferrioxamine B at seawater ionic strength," *Mar. Chem.*, vol. 173, pp. 40–51, 2015, doi: https://doi.org/10.1016/j.marchem.2015.02.014.

[49] F. J. Millero, “The Physical Chemistry of Seawater,” *Annu. Rev. Earth Planet. Sci.*, vol. 2, pp. 101–150, 1974, [Online]. Available: https://www-annualreviews-org.libproxy1.usc.edu/doi/pdf/10.1146/annurev.ea.02.050174.000533

[50] R. A. Robinson and R. H. Wood, “Calculation of the osmotic and activity coefficients of seawater at 25°C,” *J. Solution Chem.*, vol. 1, no. 6, pp. 481–488, 1972, doi: 10.1007/BF00651724.

[51] A. D. Kney and D. Zhao, “A Pilot Study on Phosphate and Nitrate Removal from Secondary Wastewater Effluent Using a Selective Ion Exchange Process,” *Environ. Technol.*, vol. 25, no. 5, pp. 533–542, May 2004, doi: 10.1080/09593330.2004.9619344.

[52] J. L. Acero, F. J. Benitez, A. I. Leal, F. J. Real, and F. Teva, “Membrane filtration technologies applied to municipal secondary effluents for potential reuse,” *J. Hazard. Mater.*, vol. 177, no. 1, pp. 390–398, 2010, doi: https://doi.org/10.1016/j.jhazmat.2009.12.045.

[53] Y. Zhao, L. Song, and S. L. Ong, “Fouling behavior and foulant characteristics of reverse osmosis membranes for treated secondary effluent reclamation,” *J. Memb. Sci.*, vol. 349, no. 1, pp. 65–74, 2010, doi: https://doi.org/10.1016/j.memsci.2009.11.023.

[54] S. Kang, K. Choo, and K. Lim, “Use of Iron Oxide Particles as Adsorbents to Enhance Phosphorus Removal from Secondary Wastewater Effluent,” *Sep. Sci. Technol.*, vol. 38, no. 15, pp. 3853–3874, Jan. 2003, doi: 10.1081/SS-120024236.

[55] A. C. Maizel and C. K. Remucal, “The effect of advanced secondary municipal wastewater treatment on the molecular composition of dissolved organic matter,” *Water Res.*, vol. 122, pp. 42–52, 2017, doi: https://doi.org/10.1016/j.watres.2017.05.055.

[56] R. Fuhrmann and A. Zirino, “High-resolution determination of the pH of seawater with a

flow-through system," *Deep Sea Res. Part A. Oceanogr. Res. Pap.*, vol. 35, no. 2, pp. 197–208, 1988, doi: https://doi.org/10.1016/0198-0149(88)90036-2.

[57] M. Chierici, A. Fransson, and L. G. Anderson, "Influence of m-cresol purple indicator additions on the pH of seawater samples: correction factors evaluated from a chemical speciation model," *Mar. Chem.*, vol. 65, no. 3, pp. 281–290, 1999, doi: https://doi.org/10.1016/S0304-4203(99)00020-1.

[58] J. M. Gieskes, "EFFECT OF TEMPERATURE ON THE pH OF SEAWATER1," *Limnol. Oceanogr.*, vol. 14, no. 5, pp. 679–685, Sep. 1969, doi: 10.4319/lo.1969.14.5.0679.

[59] C. T. Jafvert and R. L. Valentine, "Dichloramine decomposition in the presence of excess ammonia," *Water Res.*, vol. 21, no. 8, pp. 967–973, 1987, doi: https://doi.org/10.1016/S0043-1354(87)80015-5.
